# Sarcopenia: ¿Es posible un diagnóstico clínico?

**DOI:** 10.31053/1853.0605.v81.n1.42334

**Published:** 2024-03-27

**Authors:** Vanina Soledad Lerena, Elizabeth Carolina Coronello, Ilsen Catherine Torres Barrón, Sabrina Paola Lucas, Adriana Graciela Diaz

**Affiliations:** 1 División endocrinología, Hospital de Clínicas José de San Martín, Facultad de Medicina, Universidad de Buenos Aires Buenos Aires Argentina

**Keywords:** Sarcopenia, fuerza muscular, fuerza de prensión palmar, masa muscular, Sarcopenia, muscle strength, hand grip, muscle mass, Sarcopenia, muscle strength, hand grip, muscle mass

## Abstract

**Introducción:**

La sarcopenia aumenta el riesgo de caídas y fracturas, además de relacionarse con diversas enfermedades y aumento de la mortalidad. Distintos grupos de investigación proponen diferentes definiciones para el diagnóstico de sarcopenia, que incluyen la medición de la masa muscular por DEXA y la valoración de la fuerza de prensión palmar a través de un dinamómetro de mano. Sin embargo, estos 2 instrumentos no están habitualmente disponibles en los consultorios médicos.

**Objetivos:**

Determinar la utilidad diagnóstica de 2 pruebas de rendimiento físico: velocidad de marcha e incorporarse de una silla, como herramientas para la detección de pacientes con probable sarcopenia.

**Metodología:**

Se incluyeron 706 mujeres, entre 50 y 85 años, a las que se realizó las pruebas funcionales y DXA (n=376) para medir la masa muscular.

**Resultados:**

El 26,1% registró una velocidad de marcha disminuida mientras que el 55,3% presentó alteración en la realización de la prueba de incorporarse de una silla. La prevalencia de sarcopenia varió desde un 4,5% hasta un 35% según los criterios utilizados. Ambas pruebas presentaron un alto valor predictivo negativo (>70%) para el diagnóstico de sarcopenia, mientras que la prueba de incorporarse de una silla mostró además, una alta sensibilidad (> 66%) para dicho diagnóstico.

**Conclusión:**

La implementación en el consultorio de estas pruebas sencillas y sin costo, simplificaría la detección de pacientes con probable sarcopenia.

## Abstract (en)

Sarcopenia increases the risk of falls and fractures, as well as being related to various diseases and increased mortality. Different research groups propose different definitions that include the measurement of muscle mass by DXA and the assessment of hand grip strength through a hand dynamometer. However, these 2 instruments are not usually available in clinical practice. Our objective was to determine the diagnostic utility of 2 physical performance tests: gait speed and sit to stand test, as tools for the detection of patients with probable sarcopenia. A total of 706 women between 50 and 85 years of age were included and 376 of them completed the evaluation with the measurement of muscle mass. Anthropometric measurements were taken and physical performance was evaluated. We found that 26.1% registered a reduced gait speed while 55.3% presented alterations in performing the sit to stand test. The prevalence of sarcopenia ranged from 4.5% to 35% depending on the criteria used. Both tests showed a high negative predictive value (>70%) for the diagnosis of sarcopenia, while the sit to stand test also showed high sensitivity (>66%) for this diagnosis. We concluded that the implementation, in the clinical practice, of these easy and free tests would simplify the detection of patients with probable sarcopenia.

## Resumo

**Introdução:**

A sarcopenia aumenta o risco de quedas e fraturas, além de estar relacionada a diversas doenças e ao aumento da mortalidade. Diferentes grupos de pesquisa propõem diferentes definições para o diagnóstico da sarcopenia, que incluem a mensuração da massa muscular por DEXA e a avaliação da força de preensão palmar por meio de dinamômetro manual. No entanto, estes 2 instrumentos não estão normalmente disponíveis em consultórios médicos.

**Objectivos:**

Determinar a utilidade diagnóstica de 2 testes de desempenho físico: velocidade da marcha e levantar-se da cadeira, como ferramentas para detecção de pacientes com provável sarcopenia.

**Metodologia:**

Foram incluídas 706 mulheres, entre 50 e 85 anos, que realizaram testes funcionais e DXA (n=376) para mensuração de massa muscular.

**Resultados:**

26,1% registraram redução na velocidade da marcha enquanto 55,3% apresentaram alteração no desempenho do teste para levantar da cadeira. A prevalência de sarcopenia variou de 4,5% a 35% dependendo dos critérios utilizados. Ambos os testes apresentaram alto valor preditivo negativo (>70%) para o diagnóstico de sarcopenia, enquanto o teste de sentar na cadeira também apresentou alta sensibilidade (>66%) para esse diagnóstico.

**Conclusão:**

A implementação em consultório destes testes simples e gratuitos simplificaria a detecção de pacientes com provável sarcopenia.

**CONCEPTOS CLAVE**

**¿Qué se sabe sobre el tema?**

La sarcopenia es la disminución de la fuerza y de la calidad del musculo, con el consecuente aumento del riesgo de caídas y fracturas. Para arribar a su diagnóstico existen distintos criterios establecidos por consenso, pero en todos ellos es necesario realizar estudios para evaluar la masa muscular y la fuerza muscular.

**¿Qué aporta este trabajo?**

La utilización de pruebas de rendimiento físico, fácilmente realizables en consultorio para identificar a los pacientes con probable sarcopenia.

**Divulgación**

La sarcopenia es una nueva enfermedad que afecta la calidad del musculo, provocando mayor riesgo de caídas y fracturas. Hoy en día para llegar al diagnóstico y empezar un tratamiento es necesario contar con herramientas como densitómetro y dinamómetro, por lo que nuestro trabajo evaluó un gran número de mujeres para poder establecer si sólo realizando pruebas funcionales en el consultorio como velocidad de marcha y levantarse de una silla podíamos identificar a las personas con sarcopenia, de manera fácil y económica.

## Introducción

En 1989, Irwin Rosenberg acuñó el término "sarcopenia" (del griego "sarx", carne y "penia", pérdida) ^1^ para referirse al proceso de pérdida de masa y tamaño del músculo esquelético relacionado con la edad. Posteriormente, se agregó a esta definición la alteración de la función muscular ^2^ . De este modo, la sarcopenia constituye un proceso degenerativo del músculo que involucra la disminución de su masa y alteración de su función. Los pacientes que padecen sarcopenia tienen mayor riesgo de caídas y fracturas ^3^ . Desde hace muchos años, se conoce que una disminución de la carga mecánica por afectación muscular, favorece la fragilidad ósea ^4^ . Esta interacción músculo-hueso parece ser multifactorial y bidireccional e involucra no sólo la teoría del mecanostato, sino también diversos factores endocrinos y parácrinos ^5^ . Por lo que actualmente, se ha puesto la mirada sobre el músculo como un actor importante en la aparición de fracturas osteoporóticas. Se estima que 1 de cada 3 mujeres y 1 de cada 5 varones sufrirá una fractura osteoporótica luego de los 50 años ^6^ . Actualmente, diversos grupos de investigación como la United States Foundation for the National Institutes of Health (FNIH) y el European Working Group on Sarcopenia in Older People (EWGSOP), entre otros, han tratado de establecer los criterios para definir el diagnóstico de sarcopenia, pero no se ha logrado un consenso entre los mismos(^2,7,8^). Todas las definiciones incluyen la medición de la masa muscular por absorciometría dual de raxos X (DXA) y la valoración de la fuerza de prensión palmar (FPP) a través de un dinamómetro de mano. Sin embargo, estos 2 instrumentos no están habitualmente disponibles en los consultorios médicos, por lo que resulta difícil realizar el diagnóstico de esta patología en la práctica clínica diaria. De este modo, el objetivo de nuestro trabajo fue determinar la utilidad diagnóstica de 2 pruebas de rendimiento físico: prueba de velocidad de marcha y prueba de incorporarse de una silla, como herramientas para la detección de pacientes con probable sarcopenia.

## Materiales y métodos

Se realizó un estudio descriptivo de corte transversal en el Hospital Universitario de Clínicas "José de San Martín" que involucró a 706 mujeres de 50 a 85 años que acudieron a una convocatoria abierta a la comunidad para estudio de la salud ósea y muscular.

Todas las participantes respondieron una encuesta sobre factores de riesgo para osteoporosis, incluyendo antecedentes personales de enfermedades crónicas (diabetes, enfermedades reumáticas, insuficiencia renal, neoplasias malignas), hábitos tóxicos, consumo de fármacos y caídas en el último año. Se tomaron medidas antropométricas (peso, talla, IMC y perímetro de cintura). La obesidad se definió mediante el índice de masa corporal (IMC=peso/talla^2^) ≥30 kg/m^2^, según la clasificación de la OMS. Se excluyeron aquellas mujeres con artritis o dolor en las manos y enfermedades que pudieran potencialmente afectar la evaluación muscular como miastenia gravis, polimiositis, dermatomiositis, esclerosis múltiple y enfermedad de Parkinson.

Se midió la fuerza muscular por dinamómetro digital de mano Camry (participante sentada en una silla con respaldo, se evaluó el brazo dominante con el codo flexionado en 90°, sin apoyar en superficie alguna y con el dinamómetro en posición vertical, midiendo la fuerza de prensión máxima durante 3 segundos, se registró el mayor valor obtenido de dos oportunidades, con reposo de 1 min entre cada repetición), considerando un valor normal ≥20kg o ≥16kg, según los criterios establecidos por la FNIH2 y el EWGSOP2, respectivamente. Además, se evaluó la capacidad funcional mediante la prueba de velocidad de marcha y la prueba de incorporarse de una silla. La primera de ellas, consistió en una caminata recta cronometrada de 4 metros, siendo normal aquella realizada en ≤5 segundos (<0,8 m/s) ^9^ . En la segunda prueba, la participante debía sentarse y pararse de una silla, sin ayuda de los brazos, 5 veces consecutivas, estableciendo como valor normal la capacidad de realizarla en ≤11,2 segundos ^10^ . Ambas pruebas se repitieron 2 veces con un intervalo de 5 minutos y se consideró el menor tiempo obtenido.

De las 706 participantes, un grupo de 376 accedieron a completar su evaluación mediante la medición de la masa magra apendicular (MMA= masa magra de brazos + masa magra de piernas) por DXA Lunar DPX. Para el diagnóstico de sarcopenia se utilizaron las definiciones establecidas por la FNIH2 y por el EWGSOP2. De este modo, la FNIH2 define a la sarcopenia como MMA/IMC <0,591m^2^ con FPP <20kg, mientras que el EWGSOP2 considera sarcopenia a MMA/talla^2^ <5,5kg/m^2^ con FPP <16kg.

El análisis estadístico se realizó con el software IBM SPSSv.20.0. Se consideró x̄ y DS y test no paramétricos para variables cuantitativas y test de Chi^2^ para variables cualitativas. Se estableció una significancia estadística a una p <0,05.

## Resultados

El grupo de 376 mujeres estudiadas presentaba una edad de 66,1 ± 8,7 años. Según el IMC, el 35% de las participantes eran obesas (IMC≥30) y solo el 30% tenían IMC normal.

En cuanto a las pruebas de rendimiento físico, se observó que el 45,2% de las participantes presentaba una FPP <20kg y el 19,7% de ellas, una FPP <16kg. El 26,1% registró una velocidad de marcha disminuida mientras que el 55,3% presentó alteración en la realización de la prueba de incorporarse de una silla. La tabla 1 muestra los resultados obtenidos en cada una de las pruebas realizadas según los distintos grupos etarios, observándose una disminución significativa de la velocidad de marcha y de la prueba de incorporarse de una silla con la edad (p < 0,0001) y una tendencia para la prueba de fuerza de prensión palmar, aunque esta última no alcanzó significancia estadística en ninguno de los 2 puntos de corte establecidos.

**Tabla 1:** Resultados de pruebas funcionales distribuidas según grupos etarios

**n de pacientes con prueba alterada (%)**							
***Prueba funcional***	***Valor normal***	**Total** **(n=376)**	**50-59 a** **(n=89)**	**60-69 a** **(n=159)**	**70-79 a** **(n=97)**	**>80 a** **(n=31)**	**p**
**Velocidad de marcha 4 m**	≤5 seg	98(26)	15(16)	32(20)	37(38)	14(45)	<0,0001
**Incorporarse de una silla**	≤11,2 seg	208(55)	30(33)	94(59)	64(65)	20(64)	<0,0001
**Fuerza Prensión Palmar (FPP)**	≥20 Kg	170(45)	40(44)	63(39)	48(49)	19(61)	0,095
	≥16 Kg	74(19)	15(16)	27(16)	22(22)	10(32)	0,054

Al analizar la masa muscular, observamos que el porcentaje de mujeres con MMA baja dependía del criterio utilizado. De este modo, la MMA se encontraba disminuida en el 39,4% de las participantes si se ajustaba al IMC, mientras que ese porcentaje caía al 22,6% al ajustarlo a la talla. Asimismo, la prevalencia de sarcopenia varió según los criterios utilizados para su definición, siendo del 35% según la FNIH2 y del 4,5% según el EWGSOP2.

El 38% (143/376) de las mujeres refirieron haber presentado al menos 1 caída en el último año, sin diferencias de edad o IMC con respecto a quienes no reportaron caídas. Sin embargo, estas mujeres mostraron menor rendimiento físico en las pruebas realizadas. Aquellas mujeres que refirieron caídas requirieron mayor tiempo para realizar la prueba de velocidad de marcha (4.82 ± 1.8 vs 4.5 ± 1.6, p=0,049) y la prueba de incorporarse de una silla (14 ± 6.1 vs 12.3 ± 4.11, p=0,009) respecto de quienes no sufrieron caídas. Además, las mujeres que refirieron caídas presentaron menor fuerza muscular con FPP <16kg (p=0,001; OR: 2.27 IC= 1.35-3.8) o FPP <20kg (p=0,002; OR: 1.92 IC: 1.26-2.93).

Por último, evaluamos la exactitud diagnóstica de las pruebas de velocidad de marcha y de incorporarse de una silla para la predicción de sarcopenia según las definiciones de la FNIH2 y del EWGSOP2. Observamos que ambas pruebas presentaron un alto valor predictivo negativo para el diagnóstico de sarcopenia, mientras que la prueba de incorporarse de una silla mostró, además, una alta sensibilidad para dicho diagnóstico (tabla 2) tanto en el grupo de mujeres obesas como no obesas.

**Tabla 2:** sensibilidad y especificidad de las pruebas funcionales para predecir sarcopenia según FNIH2 y EWGSOP2, en obesos y no obesos

**Velocidad de marcha**	SARCOPENIA SEGÚN FNIH2			SARCOPENIA SEGÚN EWGSOP2		
	Total	Obesos (IMC ≥ 30)	No obesos (IMC < 30)	Total	Obesos (IMC ≥ 30)	No obesos (IMC < 30)
Sensibilidad (%)	38	38	39	35	0	38
Especificidad (%)	81	81	80	74	72	76
Valor predictivo positivo (%)	52	65	44	6	0	10
Valor predictivo negativo (%)	71	58	77	96	99	95
**Prueba Incorporarse de una silla**	SARCOPENIA SEGÚN FNIH2			SARCOPENIA SEGÚN EWGSOP2		
	Total	Obesos (IMC ≥ 30)	No obesos (IMC < 30)	Total	Obesos (IMC ≥ 30)	No obesos (IMC < 30)
Sensibilidad (%)	66	65	67	88	1	88
Especificidad (%)	47	41	49	44	38	47
Valor predictivo positivo (%)	39	50	33	7	1	11
Valor predictivo negativo (%)	73	56	80	99	100	98

## Discusión y/o conclusión

La disminución de la masa muscular y el incremento de la masa grasa que se producen con la edad desencadenan diversas patologías que contribuyen a la disminución de la calidad de vida. La sarcopenia, no solo se ha relacionado con caídas y fracturas osteoporóticas, sino también se ha asociado con enfermedad cardíaca, respiratoria, deterioro cognitivo, aumento del riesgo de hospitalización y de la mortalidad(^5,11^). Sin embargo, recién en el año 2016 fue reconocida como una enfermedad con un código ICD-10-MC ^12^ , aunque todavía no hay consenso en los criterios para su diagnóstico. Un meta-análisis publicado recientemente, sobre más de 690.000 individuos ambulatorios de todo el mundo, mostró que la prevalencia de sarcopenia varía ampliamente desde 0,2% al 86,5% según el criterio utilizado, los instrumentos empleados para la medición de la masa muscular y las características sociodemográficas y región geográfica de la población estudiada ^13^ . En concordancia con esto, nuestros resultados mostraron una amplia diferencia en la prevalencia de sarcopenia según el criterio y puntos de corte utilizados, siendo del 35% según la FNIH2 vs. 4,5% según EWGSOP2. En nuestro estudio, una alta proporción de las mujeres analizadas tenían obesidad, por lo que teniendo en cuenta esta circunstancia, la definición de sarcopenia que mejor se podría ajustar a la cohorte evaluada sería la establecida por FNIH2, ya que tiene en cuenta el IMC para su evaluación e identificaría a una mayor cantidad de pacientes con sarcopenia, lo que nos posibilitaría un tratamiento precoz y prevención de complicaciones, como caídas y fracturas. La disminución general de actividad física observada en los obesos podría favorecer la disminución de la fuerza muscular, además del estado pro inflamatorio típico de la obesidad y la mayor infiltración grasa del tejido muscular. La obesidad y la sarcopenia en los ancianos pueden potenciarse mutuamente, maximizando sus efectos sobre la discapacidad, la morbilidad y la mortalidad ^14^ . En nuestro estudio observamos que las pruebas funcionales permitieron aproximar al diagnóstico de sarcopenia, independientemente de la obesidad y el criterio utilizado.

El oportuno y adecuado diagnóstico de la sarcopenia es un paso importante en la prevención de caídas, fracturas por fragilidad y diversas patologías, en la creciente población de adultos mayores. Si bien en la última década, la investigación sobre el tema ha aumentado de manera exponencial, sigue siendo difícil la aplicación de los algoritmos y las definiciones diagnósticas en la práctica clínica diaria de consultorio. Se ha propuesto el cuestionario SARC-F como una primera aproximación al diagnóstico de sarcopenia ^15^ . Este cuestionario evalúa, mediante 5 preguntas, la percepción del individuo en cuanto a su capacidad de levantar peso, su deambulación, subir escaleras e incorporarse de una silla y las caídas sufridas en el último año. Por el contrario, las pruebas de rendimiento físico son herramientas objetivas para evaluar la capacidad funcional del individuo. A través de nuestro estudio hemos demostrado que las pruebas de velocidad de marcha y de incorporarse de una silla, 2 pruebas fácilmente realizables en el consultorio, permitirían identificar individuos con o sin sarcopenia, principalmente en ámbitos donde no se cuenta con un dinamómetro de mano y/o con el DXA, instrumentos sugeridos por los consensos para el diagnóstico de esta patología. Si bien, la evaluación muscular podría estar condicionada por circunstancias que se dan frecuentemente en la población de edad avanzada, como son la inestabilidad en la marcha, los problemas de visión y el dolor por enfermedades inflamatorias crónicas, estas situaciones llevan a menor movilidad lo cual contribuye al deterioro de la masa muscular. Del mismo modo, se ha reportado una mayor prevalencia de sarcopenia en enfermedades inflamatorias crónicas como la artritis reumatoide o lupus eritematoso sistémico, entre otras(16,17), aún en individuos jóvenes e independientemente del grado de actividad de la enfermedad ^18^ , pudiendo considerarse que el compromiso muscular en estos pacientes, podría ser tanto causa o consecuencia de esta enfermedad.

La implementación en el consultorio de estas pruebas sencillas y sin costo, simplificaría la evaluación de los pacientes. El aumento de detección de sarcopenia permitiría diseñar estrategias alimentarias y de modificación de estilos de vida y/o de rehabilitación, con el objetivo de prevenir y/o retrasar la aparición de esta enfermedad, mejorar la calidad de vida, mantener la independencia de los adultos mayores y reducir los costos en salud.

Este estudio tiene algunas limitaciones como ser: involucrar solamente mujeres mayores de 50 años reclutadas a través a una convocatoria abierta a la comunidad para la evaluación de su salud ósea y muscular, este método de reclutamiento podría constituir un riesgo de sesgo de la muestra y no ser representativa del resto de la población femenina. En segundo lugar, que las participantes fueron reclutadas en un único centro de salud, por lo que los resultados podrían estar influidos por una cuestión regional socioeconómica y cultural. Futuros estudios multicéntricos podrían aportar datos que abalen nuestros resultados.
